# Our Christmas Tableaux Vivants

**Published:** 1892-12-24

**Authors:** 


### Our Christmas Tableaux Vivants.

It is one of the commonest of common mistakes that all persons are always suffering who are sick. But as a matter of fact there are few more cheerful places in the world than the inside of a hospital ward. In that bright abode everything, or almost everything, requisite for happiness is found —good cheer, good service, and good company. The hospital table, with its excellent plain cooking, must be delightful after the comparatively poor and poorly-cooked food of the workman's home; the skilful doctoring and cheery help of physicians, surgeons, house surgeons, and students supply a veritable stimulus to the mind, the effect of which is to raise the spirits and rouse the interest in a very high degree; the trim nurses, with their disciplined kindness, and their breezy fashion of blowing all troubles away, complete an experience which is for the most part entirely novel, and which is almost always looked back upon by all sorts of persons as a "red letter period" in their life. "Cheer boys, cheer, no more of idle sorrow," is the kind of motto which writes itself large upon many a sick man's brain after he has been tucked up and cared for for three or four weeks in a hospital.

### Thirty Millions Sterling.

There are a great many people in London and elsewhere who never look at hospitals. Well, let us for a moment enact the part of showman for the indifferent. It is estimated that the capitalised value of the London hospitals, with their sites and other resources, is not less than thirty millions. It is not every day, ladies and gentlemen, you can see philanthropy on this scale! Above all, it is not every day the "canting philanthropist," as you amiably call him, gets the whip-hand of you, as he does when he sternly bids you look at the hospitals and admit that he can work as well as talk. Do not be afraid to look on this truly noble monument which a despised philanthropy has reared aloft. It is not a shadow; it will not melt away as you look; it is solid and substantial; its foundations are laid deep in the English character, and, depend upon it, the superstructure will rise higher and higher till, of all the memorials of our national greatness, it shall be the greatest and the most magnificent. To all that noble thirty millions you have not as yet contributed a single penny; and you have laughed at those who have. You are ashamed of yourself? That is encouraging. Laugh no more, but resolve that of the next thirty millions which hearts of honest pity raise for their poorer fellow men you will contribute at least your full quota, and even more.

### Some Human Documents.

"But how is it all spent?" "Aha! You are caught there," the snarling objectors snap out. Wait a moment, sirs, till we draw up the curtain once more. Look now at the next scene in the play! You will need all your best eyesight. Here is a display, not of property, but of humanity for you. There they come: see if you can count them! One, two, twenty, a hundred, a thousand, half a million! What! Will they never stop coming? How many of these sick and helpless ones are there altogether? Since last Christmas, good friend there have not been fewer than 962,610, all told; as nearly a million as you need care to count. And they have all been provided for, sir! Have had their bones mended when broken, as you would like to have yours mended if broken; have had their pains and agonies relieved, as you would wish yours to be relieved if you had them; have been nursed and comforted and wooed back from the very doors of death, as you would piteously ask to be nursed and comforted and wooed back if you felt yourself within a single pace of the grim portal. A round million of these poor and helpless sick, as near as may be! A million in a single year! In London alone.

It was a rare sight, that million! The writer can by no means realise it in imagination, nor, he supposes, can his readers. It needs to be broken up into pieces. Let age have precedence! The white-haired among the sufferers are not a few: tottering old men, and worn-out-looking women are very plentiful. One cannot feel harshly towards these poor old creatures. They may not have fought very victoriously in life: nay, they have not fought victoriously at all, or they would not be here. But some of them have broken limbs; some are paralyzed; some are blind; and all are helpless. God pity you old men and women. As for us—we shall do the utmost that is in us to make you at least a smooth pathway to the grave.

### Brave Britons Little and Big.

From age to youth: from the old man to the child! The poor child: the pale and underfed little one. His spine is curved, perhaps; or he has had a club foot "righted"; and there has been much cutting and pressure and pain; and his eyes look as if the tears were near the surface. But he is a brave little man for all that; an Englishman, you must remember. Moreover, he has caught the "trick" of the hospital; and the "trick" is to bear all things bravely; to be a hero when heroism is an indispensable necessity. Even those small men and women whose age is yet in single figures have learnt that what can't be cured must of necessity be endured: and they endure it—with hearts that are sometimes so stout and great that the little breasts can hardly contain them. God bless you, too, ye conscript children of an Empire which, though vast it be, is not too great to remember you, and to provide for your pains and needs! How many do you say you number? "More than a hundred thousand." What, you will tell us the exact figure—102,983! Such a chattering crowd of you in the London Children's Hospitals every year! Why, you are an army! Never mind, you will live to fight for your country some day; and if you do not fight you will work for it: and you will not work any the worse because you have had a few months of discipline and experience inside the hospital.

"Make way now for the stalwarts," is the showman's resounding cry. The stalwarts! Who are the stalwarts? What have stalwarts to do with hospitals? The stalwarts are the bread-winners, ladies and gentlemen: the bread-winners of both sexes. They are here in the hospital because some accident has smashed their limbs, or some subtle fever has taken away their strength and health, and reduced them to poverty and helplessness. Make way for these without the delay of a moment, because they are needed elsewhere, and must be cured at once. The children at home will be on short commons if you delay their parent's cure; their clothes will rush to raggedness, and the furniture may have to go to places which should not even be named in a poor man's house without a shiver. Poverty and the pawnshop: the pawnshop and deeper poverty. These are the ghosts that haunt the workman's home if you do not provide him with a hospital and hospital science, so that he may be speedily cured and promptly returned to his work. Did you ever try to imagine what an army of half a million fighting men might mean? A larger army than that—nearer a million, in fact—of men and women bread-winners are treated and cured by the London hospitals every year. As Dominie Sampson used to say, "It is 'prodigious;'" and it is even more than "prodigious," Dominie—it is magnificent.

Mr. Gladstone's "Leaders of the Future."

But a graver tone becomes the showman now. This time, when the curtain is drawn up, you will not see an army. No! not an army, but a regiment; a regiment which we have seen before, but which we are beginning to regard with a new curiosity. Mr. Gladstone tells us that the day is near when this regiment will lead the van in the world's march. Already we of the regiment seem to hear the charging order, "Up, guards, and at 'em!" The regiment is in frock-coated uniform. Bald heads and spectacles are conspicuous features. An aspect of learning is prominent, but not aggressively prominent. The most striking characteristic on almost every one of these faces is a kind of grave practicality of look and tone. An interesting sight these hospital doctors present; and there are at least seven hundred of them; seven hundred doctors voluntarily giving their services for nothing to the London poor. Do not be in a hurry to sneer, Mr. Spare-no-cash, and to tell us that these doctors expect their *quid pro quo!* What if they do! Are you yourself in the habit of giving a *quo* without expecting a *quid?* The question you should ask is this: Are the London poor benefited by doctors who take hospital service as an unpaid means of advancing their career more than they are by the paid services of State-appointed doctors? The answer is prompt and unanimous. The voluntary and endowed hospitals of this country render better service to the poor than any other hospitals which the world ha ever seen. An army, or rather a regiment of seven hundred physicians, surgeons, and house surgeons lay themselves out to serve the London hospitals as if every out-patient paid a two guinea fee, and every in patient were a prince of the blood. If you, Mr. Sparenocash, tell us that "the doctors motives are not all pure philanthropy," we answer, what have you to do with philanthropy? You never help the hospitals at all, either for your own advancement or for the public good. Instead of your miserable objections, see if you cannot find some *locus poenitentiae* from which you yourself may put away your atrabiliousness and make beginning of a kindlier life!

"A Dream of Rare Women."

But a truce to sharp speeches! Another tableau awaits us. Concerning this we hardly know what to speak. It is a tableau of women; of fair women and dark women; of tall women and dumpy women; of pretty women and—women who would, no doubt, be pretty if their uniforms were a little more becoming. For not all nurses uniforms are becoming, and not all nurses are becoming in their uniforms. But it is a brave sight; it is a regiment of Amazons; not physical Amazons, but mental and moral strong women, and brave battlers against all sorts of ills. There are a thousand of these trained and disciplined soldiers of the gentler sex, at the very least, a thousand efficient nurses working in the London hospitals alone, many of them of the lady probationer class, receiving no wages at all, and some of them paying out of their own pockets for the valuable training and discipline they are so rapidly acquiring. The trained nurse, although she has been a little spoilt lately, is a quite indispensable institution. She is something like the little girl in the nursery rhyme, "When she is good, she is very, very good; and when she is bad, she is horrid." But she is generally good; we insist upon that. And think of the work which a thousand well-trained and competently-officered women can do in a single day, in six, nay, seven days a week. All this work is at the service of the deserving London poor from the beginning of the year even unto the end of the year. What an army these thousand women, with their seven hundred black-coated guides and coadjutors of the medical profession constitute! This is what your money does, ye kindly philanthropists! It employs these one thousand seven hundred highly-trained men and women all the year through, in the service of the industrious and deserving poor.

A Glance at the Day's Work.

That is a glance, a mere momentary glance, at the hospitals and their patients and workers. Let us now see a little of their work. The public, and even the newspapers, were quite startled last Christmas when we informed them that there were at least 250,000—a quarter of a million—accident cases of greater or less severity treated at the various London hospitals every year. Guy's alone treated over 21,000, and performed no fewer than 1,200 "major," or greater, operations. We have seen the patients, doctors, and nurses in the mass, and those were sights to be remembered. But now we have to think of every individual physician at the bed-side in his own ward; and of every individual surgeon with his coat off, his sleeves tucked up, and a linen apron on, performing operations in which are involved the issues of life and death. Seven hundred of these, separately at work, with their attendant nurses and students every day! That is what London philanthropy, despised philanthropy, is doing. Will not every honest English heart say firmly, and even proudly, "If that be philanthropy, let me be a philanthropist, though all the geese on all the commons in the world should cackle at it!"

The Motive Power.

We are well-nigh forgetting to say anything about the money aspect of all this work, so entirely full of pride and satisfaction are we with the work itself. But no mill can grind without corn, or rather without water, or wind, or steam power. The corn we have in too great abundance in the form of helpless patients. The motive power, though great, is all too small for the vast amount of work to be done. There are 119 hospitals in and around London, all of which, with perhaps the solitary exception of St. Bartholomew's, could do more work if they had more money. And the work is there to be done; and much needless suffering and loss of life are the result of its not being done. This is a bare fact, without either exaggeration or pleader's rhetoric. We said a little while ago that the sum of nearly £600,000 is spent annually upon all the London hospitals; and this seems to us an astonishingly small amount when we consider that it commands the best services of 700 of the most accomplished of London doctors all the year round; of more than 1,000 trained nurses; of innumerable servants, washers, scrubbers, and what not; and that it feeds and houses nearly 100,000 in-patients; and that it supplies with medicines and surgical instruments 900,000 more. This, we say, is an immense series of striking and worthy effects from a decidedly modest cause.

For years we have been trying to make the public see and understand this question as it really is; and we begin to feel that at last there is hope of great and enduring success. The only absolute success would, of course, be that the income of the hospitals should balance their expenditure every year, and that there should be an expanding subscription and legacy list which would keep pace with the growing needs of an increasing population. This degree of success has not been reached yet; and there are people who think it never will be reached. But we are fully convinced that it will be reached if the character of Englishmen continues to improve, and the resources of England continue to expand. One question, which we often ask ourselves, seems to throw a a good deal of light upon the subject. The question is this: Have the hospitals exhausted all the arts of instructing the public and convincing it of the value and indispensable necessity of their existence and work? For our part, we are satisfied that the arts of mere begging have been thoroughly exhausted; and we are thankful for it. But instruction—candid, manly, intelligent instruction—has hardly so much as begun. Englishmen are not fools, though there are plenty of fools among them; and the majority of newspaper writers hate exaggeration and manufactured scandal with all their hearts. Hospitals have to recognise that, together, they constitute a sort of small kingdom, and, to a certain extent, an unknown, or, at best, half understood, kingdom. In these circumstances, if they wish to live by the voluntary support of the public, they must make the public thoroughly acquainted, not only with their wants, but also with their works and ways. It is our experience that Englishmen cannot help giving when they are fully enlightened and convinced of the worthiness of the cause they are asked to support. The great need of the moment, therefore, is instruction. Let the hospitals for a brief period devote themselves, in combination, to the better instruction of the public. Do not "beg, beg, beg," until all the world is weary of your begging; but "instruct, instruct, instruct," until every right-thinking man in the country shall run to you with his gifts, and ask you to put them for him to the noblest of all uses.

